# The AvecNet Trial to assess whether addition of pyriproxyfen, an insect juvenile hormone mimic, to long-lasting insecticidal mosquito nets provides additional protection against clinical malaria over current best practice in an area with pyrethroid-resistant vectors in rural Burkina Faso: study protocol for a randomised controlled trial

**DOI:** 10.1186/s13063-015-0606-4

**Published:** 2015-03-25

**Authors:** Alfred B Tiono, Margaret Pinder, Sagnon N’Fale, Brian Faragher, Tom Smith, Mariabeth Silkey, Hilary Ranson, Steve W Lindsay

**Affiliations:** Centre National de Recherche et de Formation sur le Paludisme (CNRFP), Ouagadougou, Burkina Faso; School of Biological Sciences and Biomedicine, Durham University, Durham, UK; Medical Research Council Unit The Gambia, P.O. Box 273, Banjul, The Gambia; Liverpool School of Tropical Medicine, Liverpool, UK; Swiss Tropical and Public Health Institute, Basle, Switzerland

**Keywords:** Insecticide resistance management, Malaria control, Insecticide-treated bed net, Pyrethroid, Pyriproxyfen, Insect juvenile hormone mimic, Clinical malaria, Entomological inoculation rate, Cluster randomized controlled trial

## Abstract

**Background:**

Recent reductions in malaria in sub-Saharan Africa have been associated with increased coverage with long-lasting insecticidal nets (LLINs). Pyrethroids are currently the only insecticide class used for treating nets, and the rapid increase in resistance to pyrethroids in vector mosquitoes may jeopardise future vector control. Nets containing a novel combination of permethrin, a pyrethroid, and pyriproxyfen, an insect juvenile hormone mimic, (PPF-LLIN) may enhance malaria control, as well as reducing the spread of pyrethroid-resistant mosquitoes. This trial will determine whether PPF-LLINs provide incremental protection against malaria over current best practice of LLINs and prompt treatment in an area with pyrethroid-resistant vectors.

**Methods:**

A 2 armed cluster-randomised controlled trial will be conducted in Burkina Faso to assess whether PPF-LLIN (containing 2% permethrin and 1% pyriproxyfen w/w) provide better protection against clinical malaria in children than 2% permethrin-treated LLINs. Study subjects will be recruited and provided with LLINs at the start of the study. The LLINs will be exchanged for PPF-LLIN by cluster in a step-wedge fashion so 3 months before the end of the 2 year trial all participants will have a PPF-LLIN. The primary endpoint will be clinical malaria incidence measured by passive case detection in a cohort of children, aged 6 months to 5 years. Anaemia and parasite prevalence will also be measured in children during cross-sectional surveys. Exposure to malaria parasites will be assessed using light traps followed by identification of common vector species and their sporozoite infection rates. Safety evaluation will include recording of adverse events and pregnancy outcomes. The main endpoint analysis will include adjusting for distance between village clusters with different types of nets, as the impact of PPF-LLIN is likely to increase as larger areas are dominated by PPF-LLIN, reducing the spill over of mosquitoes from villages with LLINs.

**Discussion:**

The step-wedge design is to measure the impact of an incrementally delivered environmental intervention where we expect the degree of control to be improved as more people use PPF-LLIN over a larger area. Trial findings will help inform policy makers on the effectiveness of PPF-LLIN nets for malaria control in areas of pyrethroid resistance.

**Trial registration:**

ISRCTN21853394 – AvecNet, registered on 3 April 2013.

**Electronic supplementary material:**

The online version of this article (doi:10.1186/s13063-015-0606-4) contains supplementary material, which is available to authorized users.

## Background

Enormous progress has been made in the control of malaria in sub-Saharan Africa over the past decade [1,2]. A significant part of this achievement has resulted from a large-scale increase of long-lasting insecticidal net (LLIN) distribution and indoor residual spray campaigns, both interventions being extremely successful tools for malaria control [3,4]. Nonetheless, the future success of these programmes is threatened by the development of insecticide resistance in the vectors [5]. Resistance is of particular concern since, until now, bed nets can only be treated with pyrethroids because of their rapid knockdown activity, high mosquito mortality ability and extremely low toxicity to humans [6,7]. Thus, although an intact bed net is protective as a physical barrier [8,9], tolerance of malaria vectors to pyrethroids would dramatically reduce the protection afforded by LLINs.

In order to increase the effectiveness of LLINs, net treatments are being developed where nets are treated with a pyrethroid in combination with another active ingredient, such as pyriproxyfen [10,11]. Pyriproxyfen is an insect juvenile hormone mimic that is recommended for vector control by the World Health Organization (WHO) because it is effective at extremely low concentrations, is safe for people [6], and has no cross resistance with other classes of insecticide used for vector control. Its primary effect is to prevent metamorphosis of pupae into adults. In addition, it can sterilise adult mosquitoes, or at least reduce their fecundity and longevity [10,12-15]. Pyriproxyfen has good residual activity in fresh water, with studies on mosquito control showing that it can be effective for 5 to 9 months after initial treatment [16,17].

Polyethylene nets treated with a combination of permethrin and pyriproxyfen (PPF-LLIN; trade name Olyset Duo (Sumitomo Chemical, Japan),) have been shown to induce higher mortality and reduce blood feeding of pyrethroid-resistant *Anopheles gambiae* s.s. than permethrin-only LLINs (Olyset) in experimental hut trials [11]. However, no controlled study demonstrates that PPF-LLIN will provide incremental protection against malaria beyond the current best practice of LLIN and prompt treatment in a controlled trial. Building on current best practice, the present trial aims to measure the incremental impact of PPF-LLIN on clinical malaria in communities using LLINs alone and having access to rapid treatment. To achieve this we will conduct a cluster randomised controlled trial in rural villages to the south-east of Banfora town in Burkina Faso, a country where malaria is relatively stable [18]. Mosquitoes in this area are currently increasing in resistance to pyrethroids. In 2010 in Tiefora, located at the edge of our trial zone, there was resistance to 1,1,1-trichloro-2,2-di(4-chlorophenyl) ethane (DDT) (12% mortality), permethrin (48% mortality) and deltamethrin (39% mortality) (S N'Fale, personnel communication).

Here we describe the proposed trial design and the methodology used. Although some of the outcome measurements and methods in this protocol are based on a previous vector control trial conducted in West Africa [19], the nature of the intervention and setting have led to many novel features.

### Study objectives

#### Clinical

##### Primary objective

To assess whether PPF-LLIN provide added protection against clinical malaria in children compared with pyrethroid-only LLINs over 2 malaria transmission seasons of follow-up.

##### Secondary objectives

ᅟ

### Efficacy

- To assess whether PPF-LLIN provide added protection compared to LLINs against anaemia and/or parasite prevalence in children.
- To assess and compare the prevalence of microscopy-confirmed gametocyte carriers in the LLIN group versus the PPF-LLIN group.

### Safety

- To assess whether PPF-LLIN have a safety profile comparable with conventional LLINs in the trial population.

#### Entomological

##### Primary objective

To assess whether PPF-LLIN reduce the entomological inoculation rate (EIR) of mosquitoes collected inside houses compared to LLINs.

##### Secondary objectives

- To assess whether PPF-LLIN reduce pyrethroid resistance in the vector population.
- To assess the impact of PPF-LLIN on *Anopheles gambiae* and *Anopheles funestus* on egg production and the survival of the larvae that emerge from these eggs.

## Methods/Design

### Study area and participant eligibility

The study site is situated south of the road from Banfora and Sideradougou (10° 56’ 00” N, 004° 46’ 00” W) and is approximately 1,250 km^2^, bisected by a river in a north-south axis. It is an area of West Sudanian savannah and the climate consists of a rainy season from May to October with little rain in other months. This defines the seasonal malaria transmission with most malaria episodes experienced during or immediately following the rainy season. The study area has 6 health centres and every village is affiliated to only 1 health centre. A baseline demographic survey conducted in July 2013 enumerated 63,903 individuals living in 93 villages, with most people living in small rural villages in houses made with mud or cement walls and thatched or metal roofs. The study’s field station is based at Banfora town which is the main town in the district. This study is in an area of cotton growing since 1968 with both the S and M forms of *An. gambiae* s.s. [20].

Each village or groups of neighbouring villages with more than 50 children aged 6 months to 5 years old will be invited to join the study, and will constitute a single cluster. Following local custom, sensitisation will start by discussions with community elders and then with representatives of the whole study community in order to explain the nature and requirements of the study. Village level permission will then be sought after sensitization meetings attended by village community leaders and health staff at the health centres; attendees’ names and village of residence will be documented. Witnessed written consent will also be sought from each net recipient, before net donation and exchange (Additional file 1). During these procedures it will be made clear that people will be able to leave the study at any time but that their original net will not be returned.

A cohort of children will be enrolled to assess the impact of the intervention on malaria. A minimum of 40 children per cluster (range 40 to 75), depending on cluster size, will be randomly selected, stratified by age and invited to participate in the clinical and travel/residence surveys and passive case detection (PCD). The random sample will be stratified by age so that approximately equal numbers of children below and above 36 months will be invited into the study. This was done in case there were marked differences in clinical malaria associated with the different age groups and to ensure balance between the 2 study arms. No distinctions in the sampling scheme will be made regarding gender, ethnic group, medical condition or physical health. Children will only be included on the condition that their carers/parents give witnessed informed written consent. Assurance will also be sought from carers/parents that they expect that the child will remain resident in their village during the transmission season and will not be sent away. If informed consent is not provided for a randomly selected child then replacement children will be randomly selected from those remaining in the village.

The impact of the intervention on the density of malaria vectors and their infection rate with malaria sporozoites will be measured in each village cluster. In each cluster 6 compounds will be selected randomly, from those that have consented to join the study and have at least 4 houses. In each household a room with a single adult male sleeper will be selected if the room owner/sleeper provides witnessed, verbal consent.

### Design

**Figure 1:**
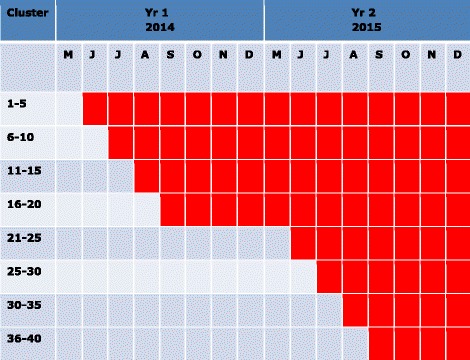
**Step-wedge design for roll-out of bednets.** Permethrin-treated nets were given to all clusters in May 2014, followed by pyriproxyfen & permethrin treated nets (red) being rolled-out to different clusters in 2014 and 2015.

A cluster-randomised controlled trial design will be used since: i) LLINs are a community-level intervention; and ii) the village cluster is a suitable unit for randomization since they represent a discrete spatial cluster. At the outset of the study, all households will be provided with new LLINs at the rate of 1.1 nets for every 2 people in their household; this will approximate 1 for every sleeping place. Each month for the next 4 months after LLIN donation, residents in 5 randomly selected village clusters will be asked to exchange their LLINs for PPF-LLIN. Stratified, random sampling will be used to ensure that by the end of year 1 (September 2014) there will be a similar number of residents with and without PPF-LLINs affiliated to each health centre. Every month from June to September in 2015, an additional 5 village clusters will be provided with PPF-LLIN, thus by the end of September 2015 all clusters will have PPF-LLIN (Figure 1). It is important that within the study area there are no villages that are not enrolled in the study in order for the PPF-LLIN to have maximum impact on larval populations and minimize the spill-over of mosquitoes from villages without these nets. LLINs and PPF-LLIN will be the same shape, size and colour and marked with a unique code number, so that the investigators are blinded to the identity of the net (apart from those field assistants distributing nets).

Clinical malaria will be recorded by study nurses using PCD methods during the rainy seasons in years 1 and 2. Parasites will be detected by rapid diagnostic tests (RDTs) and thick smears will be stained for confirmation and parasite counting by study staff. Treatment will follow government guidelines.

The study cohort will be surveyed for malaria indices (parasite prevalence, anaemia and spleen rate) at the beginning and end of both transmission seasons to generate data for the secondary endpoints. Where possible, at each malaria survey except the first, an average of 50 children from each village cluster who have not been enrolled in the cohort but are of a similar age will also be surveyed for these malaria indices. This additional group of children may better reflect the *Plasmodium falciparum* parasite rate in the population than children in the PCD cohort. Baseline data from the cohort will be used to compare the village clusters before the interventions, and the cohort from each village cluster provides its own baseline data before net exchange to PPF-LLIN. In addition, we will request anonymized medical records from the 6 health facilities located in the valley and use these to document clinical malaria in children aged 6 months to 5 years living in villages outside the study area. These children represent a further control group which can be used to track temporal changes in malaria incidence during the study period. If malaria declines in the study area in year 2, but not outside, we can be confident that the decline is due to the intervention, rather than confounding factors such as low rainfall. The end-of-season survey data will also be used to attempt to define a *P. falciparum* parasitaemia cut-off giving optimum sensitivity and specificity for malaria cases in this setting [21].

**Figure 2:**
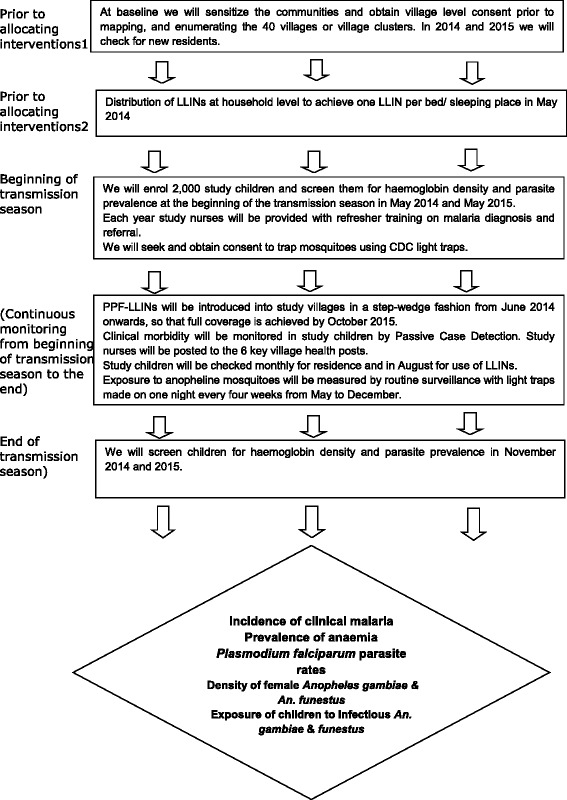
**Schematic representation of the trial.**

A schematic representation of the trial is shown in Figure 2.

### Randomisation

In cluster-randomised controlled trials it is particularly important to minimize imbalance for factors known to be highly correlated with the disease outcome - in this case age and location. In each cluster, children will be enrolled with equal numbers of children aged 6 to 35 months and 36 to 60 months, and children who drop out of the study in year 2 will be replaced, as far as possible, by children of a similar age. At each step of the wedge, clusters will be selected, as far as possible, to maintain an even balance of children in the 2 age groups. Balanced randomization will be used to ensure that, within each health centre catchment area, half of village clusters will have PPF-LLIN and half LLINs by the end of July 2014.

Stratified randomisation of village-cluster to the trial arms, as outlined above, will reduce some imbalances between these but, as only a relatively small number of units can be randomized in such a cluster design, it is important to assess baseline levels of malaria and known confounders. Baseline data parasite rates and anaemia collected from enrolled children in each cluster in May 2014 will be used to assess imbalances in malaria at the village-cluster level.

Observer bias will be reduced where feasible. Both types of nets will be of a similar shape and colour. As well as using RDTs, blood films will be read by microscopists blinded to the identity and intervention status of the subjects. Mosquito collector bias will be reduced by using standard Centers for Disease Control and Prevention (CDC) miniature light traps, which do not rely on the ability of the fieldworker to collect specimens. Trap catches will be examined by a different person to the trap collector and they will be blinded to the trap location and intervention status. The processes of the intervention, both for PPF-LLIN and LLINs, will be monitored closely not only for quality but also to document any bias between the clusters.

### Interventions

#### Long-lasting insecticidal nets

The LLINs are manufactured by Sumitomo Chemical (Japan). These are WHO recommended and meet WHO specifications with 2% w/w permethrin incorporated into polyethylene fibres giving adequate release of permethrin for about 5 years. These nets will be distributed at the beginning of the main transmission season, at no cost to the recipients. We will use white ‘extra family’ size rectangular nets (180 cm wide × 190 cm long × 150 cm high) of a white colour throughout. Government Roll Back Malaria information, education and communication procedures will be followed to encourage correct net use and maintenance, including supplying the nets in individual pre-opened bags.

LLINs will be stored locked at ambient temperature in Banfora, under the control of a delegated individual, ready to be moved to the Health Facilities in batches by lorries and held by the officers in charge before being transported to the villages for distribution by the study team. In advance of distribution, the number of beds/sleeping places and the number with LLINs will be counted. New nets will be exchanged for old nets at the household level to achieve one LLIN per bed/sleeping place. Net use by study children will be monitored during the monthly travel/residence surveys during the 2014 and 2015 transmission seasons.

#### Permethrin and pyriproxyfen long-lasting insecticidal nets

PPF-LLIN will be provided by Sumitomo Chemical (Japan) and are currently undergoing evaluation by the WHO Pesticide Evaluation Scheme. The nets contain 2% w/w permethrin and 1% w/w pyriproxyfen incorporated into polyethylene fibres giving adequate release of permethrin and pyriproxyfen for an estimated 3 years (decay rates of the insecticides on PPF-LLIN are similar to those of LLINs over 6 months, unpublished data). The PPF-LLIN will be the same size and colour as the LLINs and will be distributed the same way, but this distribution will be to the first 5 village clusters 1 month after the LLIN distribution and will be rolled out in a wedge-shaped design at monthly intervals during the main transmission season in 2014 and 2015. PPF-LLIN will be stored and distributed in a similar manner to the control LLINs.

### Clinical data collection and patient treatments

PCD for malaria will be maintained during the transmission seasons in 2014 and 2015 (June to December 2014, and June to December 2015). Parents/guardians will be encouraged to take their child to the health facility their village is affiliated with at any time their child becomes unwell. The cost of travel and treatment for sick children will be met from the study funds. Study nurses will work in close collaboration with the government health workers to monitor malaria cases as indicated above. Project fieldworkers will visit each nurse regularly, at least once a week, to record children with malaria episodes.

The end of transmission season survey in December 2015 will be the final study visit for the enrolled children in the cohort and will be similar to the end transmission season survey in 2014.

During the course of the study participants are free to receive medication from health personnel outside the study team but they are encouraged to use their allocated health facility. During the clinical surveys, sick children, those with anaemia (haemoglobin (Hb) <9 g/dL), diagnosed malaria or any other mild illness will be referred to their health facility. Children with severe malaria, or other serious conditions, will be referred to Banfora or Sideradougou Health Centres for treatment.

For safety considerations (see section below) we will identify asthmatic residents in study villages by name, household and village. During distribution and follow-up of PPF-LLIN in 2014 and 2015 these residents will be checked weekly by trial nurses for any aggravation of their symptoms for the first month after net donation and at the end of this month they will ask these residents to contact the study team if they have cause for concern. We will also identify pregnant women in study villages from antenatal clinic records, from June 2014 onwards, and outcomes of pregnancies will be recorded onto data forms, including miscarriages, live/still birth, weight and any recorded malformations. The regular monthly demographic surveillance system (DSS) in trial villages will also include reports of miscarriages. Data on asthmatics and pregnancy outcomes will then be linked to the date of reception of PPF-LLIN to examine if the events are associated with PPF-LLIN.

### Clinical evaluations

The main morbidity outcome will be incidence of clinical disease assessed by PCD; data from this will provide the primary endpoint of the study. Mild malaria and parasitaemia is common in all children in this area of highly seasonal malaria [21-23]. Study nurses in the 6 health facilities (Tiefora, Koflandé, Boussara Brousse, Madiasso, Soukara II and Kangounadeni) within the study area will document episodes of malaria in study children who present directly to them. At the health facilities malaria diagnosis will rely on the use of RDT but, in addition, thick blood films will be made for Giemsa staining and later microscopy to estimate parasite density. To facilitate documentation of all consultations by study children, all enrolled children will be issued with enumerated photo identity cards. The child’s study number, initials and village code will be recorded for all clinical events on the case report form (CRF); to maintain confidentiality the initials will not be entered into the data set.

During the surveys all children enrolled in the cohort who are present and the randomly selected children for the cross-sectional surveys will be clinically examined for obvious symptoms and signs of illness, temperature will be measured with calibrated digital thermometers and spleen enlargement will be measured by experienced nurses using Hackett’s scoring system. All children will be finger pricked for immediate measurement of anaemia using a spectrophotometer (HaemoCue®, Ängelholm, Sweden) and preparation of thick smears. If a child reports with fever in the last 48 hours and/or with a temperature ≥37.5 °C they will also be checked for presence of parasites by RDT.

The primary endpoint selected is the definition of malaria used commonly in West Africa in research studies and in National Treatment Guidelines [24-26]. This endpoint does not include a measure of parasite density and a parasite cut-off level may increase specificity. We thus also intend to use the logistic regression method of Smith and colleagues [26] to examine the effect of parasite cut-off on the specificity and sensitivity of the definition of clinical malaria. This method has been endorsed by a WHO study group for use in studies defining malaria vaccine efficacy [21].

### Entomological evaluations

Exposure to mosquitoes will be measured by surveillance with light traps to obtain the primary entomological endpoint [27]. A CDC miniature light trap will be positioned close to a single sleeper protected with a LLIN or PPF-LLIN. Mosquitoes will be identified by microscopy and the numbers of *An. gambiae* s.l., *An. funestus* and other anophelines recorded. The presence of sporozoites in *An. gambiae* s.l. will be identified using an enzyme-linked immunosorbent assay [28] and *An. gambiae* s.l. females will be typed to species by PCR [29]. In the case of *An. gambiae s.s.*, S and M forms will be identified by PCR [30]. Published kdr resistant markers will be typed by PCR [31,32].

6 sentinel clusters will be selected for measurement of the secondary entomological endpoints. Larvae will be collected from a range of breeding sites within each of these sites at the beginning and end of the transmission season in each year of the study. The prevalence of resistance to pyrethroids will be measured using standard diagnostic dose assays with papers impregnated with permethrin, obtained from WHO [33]. To quantify the strength of resistance, a modification of the CDC bottle bioassay will be used in which 3- to 5-day old non-blood-fed female mosquitoes are introduced into 250 mL glass bottles pre-coated with a range of permethrin concentrations. After a 1 hour exposure the mosquitoes are transferred to a holding tube, provided with sugar and water, and mortality recorded 24 hours later. The concentration required to obtain 50% mortality, 24 hours post-exposure is recorded.

To assess the impact of PPF-LLIN on *Anopheles gambiae* egg production and the survival of the larvae that emerge from these eggs, blood-fed mosquitoes will be collected from inside houses via manual aspiration or when exiting houses using exit traps. The female mosquitoes will be transported to the local insectary at Banfora and the number of females that lay eggs, number of eggs hatching and survival to L2 larval stages will be recorded. This will be performed in all 6 sentinel clusters at the beginning and end of the transmission season in each year. In the first round of monitoring, all houses will have standard LLINs and at the final round of monitoring all will have PPF-LLIN.

### Study procedures and evaluations

LLIN distribution will be completed in mid-2014. Study nurses and field assistants will be provided with refresher training on diagnosis of malaria, the use of RDT and study procedures in 2014 and 2015 before the transmission season.

The compound of each study child will be geo-referenced using a hand-held global positioning system, as the primary data analysis has a spatial component. Travel/residence of cohort children will be assessed at monthly intervals on brief questionnaires to estimate their time at risk, and their bed net usage will be assessed at the same time.

Thick blood films will be stained with Giemsa and examined under 1000-fold magnification by trained, experienced microscopists. Parasite counts will be recorded per high power field and 100 fields will be counted before a slide is declared negative. Parasite density will be estimated assuming that one parasite per high power field equals 500 parasites/μL [34]. 2 slides will be prepared from each subject and read separately by 2 experienced microscopists; discrepancies in positive and negative reads and those greater than 10-fold between the 2 reads will be resolved by the microscopy supervisor. Field staff will deliver all CRF to the study physician within 2 days of the clinical attack or survey date. The study physician will check all CRFs weekly for completion and data consistency.

### Safety considerations

Although adverse events (AEs) arising from either net are highly unlikely, we will document their safety in a community setting, by recording AEs and serious adverse events (SAEs) in the study cohort and population for the duration of the study. The trial will follow standard definitions for AE and SAE agreed by consensus of the Collaborating Centres of the WHO International Drug Monitoring Centre (Uppsala, Sweden). The occurrence of AEs will be sought by non-directive questioning of the study children at monthly visits during the trial. AEs will also be recorded if volunteered by study children or their parent/carer or are noted by nurses through physical examination, laboratory test, or other assessments at any contact with the participant; these will be recorded on the child’s CRF for that visit. All AEs such as skin rashes, respiratory problems, streaming eyes, and so forth, will be recorded with the grade of severity (mild, moderate or severe).

In addition, we will record pregnancy outcomes for all resident pregnant women, and asthma signs/symptoms in pre-identified asthmatic residents, as indicated in the clinical data collection and patient treatments above.

During the course of the study, SAEs will be captured using a specific SAE report form and within 2 days of the start of the SAE, field staff will deliver a completed SAE report to the study physician. The study physician will log these in a query book noting the child’s study identification number or the residents demographic surveillance system number and village of residence and report these to the Principle Investigator who will inform the responsible member of the trial Data and Safety Monitoring Committee. Excessive grouping of SAEs by clusters will be reported to the study’s Data Monitoring and Ethics Committee and Trial Steering Committee.

### Handling of drop-outs/withdrawals

If an individual wants to terminate his/her participation, no further follow-up will be performed. There will be no replacement during the surveillance period in either year but in May 2015 children who have withdrawn, or are no longer in the study for whatever reason, will be replaced by children resident in the same cluster and of the same age group if informed consent is provided. If a household withdraws consent, no further follow-up will be made in that household. A child who was participating from that household will be replaced from within the same cluster and age group and, if the house was participating in the entomology collections, it will be replaced by a neighbouring house of the same type if possible. If a village opts out of the study before July in either year replacement by a neighbouring village will be considered.

### Study endpoints

#### Clinical

Primary: incidence of clinical episodes of malaria presenting at health facilities defined as a child with an axillary temperature ≥37.5°C or a history of fever in the past 48 hours, together with the presence of *P. falciparum* parasites of any density detected by RDT in the absence of other detectable cause of fever.

Secondary: (i) mean Hb concentration in children in the 2 study arms measured at the end of the transmission season survey; (ii) *P. falciparum* parasite rates in children in the 2 study arms measured at the end of the transmission season.

In addition we will also report

- the incidence of malaria defined by history of fever or temperature ≥37.5 °C and a *P. falciparum* parasitaemia cut-off to give optimum sensitivity and specificity in this setting
- the number of children with enlarged spleens at the end of each transmission season.

#### Entomological

Transmission parameters in the 2 arms of the study will be estimated from measurements made throughout the 2 transmission seasons.

Primary: the EIR for *An. gambiae* s.l. and *An. funestus*/light trap/night inside sleeping rooms.

Secondary: (i) change in the prevalence (% mortality using diagnostic dose) and strength (LC50 value) of resistance to permethrin in each study arm throughout the study; (ii) the fertility and fecundity of adult female *An. gambiae* s.l. collected inside houses in both study arms.

Tertiary: (i) the relative abundance of different anopheline species and forms in light traps collected in each arm of the trial; (ii) the parity of *An. gambiae* to measure mean population longevity.

### Sample size rationale

#### Clinical

LLINs reduce malaria morbidity by approximately 50% [3]. The combined impact of using permethrin, a pyrethroid, and pyriproxyfen is unknown. If the effects were simply additive a mean reduction of 75% could be anticipated for PPF-LLIN (that is, LLINs alone will reduce incidence by 50%, and if we assume the pyriproxyfen alone may reduce the remaining 50% incidence by half, there would be a 75% reduction). However, the effects may be synergistic (for example, by having a mass killing effect). In order to change national guidelines on the use of LLINs we consider that PPF-LLIN should be at least 25% more effective than LLINs. A study of 3- to 60-month old children in Burkina Faso reported an incidence of clinical episodes of malaria of 1.5 episodes/child/year. Malaria incidence data for 2012 from villages in the Banfora region of Burkina Faso indicate the coefficient of variation (CV) for this study will be approximately 0.25. Regarding this study as a simple cluster-randomised controlled trial, assuming CV = 0.25 and that an average of 50 children will be monitored in each cluster for 2 seasons, the study will have 90% power to detect a 25% increase in protection with PPF-LLIN compared to LLINs (that is, a reduction in the incidence of clinical episodes of malaria from 1.5 to 1.125 episodes/child/year) at the 5% level of significance if there are 19.38 clusters in each arm [35]; we thus propose to recruit 20 village clusters into each arm of the study. Adjusting for a coefficient of up to 0.5 for the differences in cluster size (the data from Banfora cited above suggest that this CV will be much lower - approximately 15%), 20 clusters per study arm will still provide the study with 85% power [36].

Assuming prevalence of *P. falciparum* around 30% [37], 20 clusters in each arm and CV = 0.3, the study could detect a 30% reduction of prevalence, with 5% significance and 80% power, between the study arms if longitudinal cohorts for each cluster contain at least 45 children. Assuming mean Hb around 10.5g/dL, 20 clusters in each arm and CV = 0.1, the study could detect a difference of 1.0g/dL in mean Hb, with 5% significance and 80% power, between the study arms if at least 35 children were followed in each cluster .

#### Entomological

Based on a study using light traps in an adjacent study area in Burkina Faso in 2010, we expect that the mean number of female *An. gambiae* s.l. per trap/village will be 45 during the rainy season (with a CV = 0.35). In order to demonstrate a 33% reduction in blood-questing indoor mosquitoes (that is, unfed *An. gambiae*) associated with PPF-LLIN, with 80% power and at the 5% level of significance, would require 6 houses in each cluster and 14 clusters in each arm of the trial over 2 years [35]. To allow for loss to follow-up due to people moving house during the study period we propose to include at least 6 houses in each cluster and 20 clusters in each study arm for 2 years.

### Data handling and record keeping

The demographic data will be recorded by fieldworkers and the clinical data by study nurses on standardised data forms. Each child in the cohort will be identified by a unique identification number and non-cohort children participating in the cross-sectional surveys will be identified by their demographic surveillance number. All forms and datasets will identify participants by these numbers, and names will not be entered.

After the collection and verification, data will be moved to the data management unit in Banfora for data entry. Data forms will be double entered (firstly by field staff and then by data entry staff) and the entries combined and errors corrected to produce a single dataset. This will be checked for consistency by generic and study-specific algorithms designed to identify sources of error. Inconsistencies will be checked against the original forms and errors will be corrected where possible, for example by checking in the field, or put to missing, to produce final datasets.

All forms with subject names and/or clinical data will be kept in a cabinet and locked when not in use; the key will be kept by the study coordinator or delegate. The electronic datasets will be password protected. Hard copies of the data will be stored for 10 years. Clinical forms will be kept separately from that containing personal identification (for example, consent forms). The datasets will be password protected and only accessed by authorized study staff and the study data manager until after publication. Data will be stored for at least 10 years.

### Analytical plan

A per protocol and an intention to treat analysis will be conducted.

### Outcome 1 – malaria morbidity

The primary endpoint is a comparison of the incidence of clinical episodes of malaria in children in the 2 intervention groups, measured by PCD. After a case of malaria is treated, time of observation will be censored for 3 weeks and further attacks of malaria during this period are not considered. History of travel away from the village for periods of over a week will be captured by the monthly surveys and time at risk will be censored for such periods. In addition, malaria cases in children who resided outside their study village for more than half the elapsed study period at the time of illness will be censored. An initial analysis will compare the incidence rates between the 2 intervention groups, adjusted for duration of sleeping under a PPF-LLIN. However, the main endpoint analysis will also adjust for distance between village clusters with different types of nets [38]. This adjustment is critical since the impact of PPF-LLIN is likely to increase as they cover larger areas and so reduce the spill over of mosquitoes from villages with LLINs. Specifically, a village with PPF-LLIN surrounded by other villages with PPF-LLIN is likely to be better protected against malaria than a village with PPF-LLIN surrounded by villages with LLINs. Since the replacement of drop-out individuals may introduce bias we will repeat the primary analysis including and excluding this group.

Each of the outcome measures collected in this study will be analysed using multi-level statistical modelling methods, with appropriate distributional assumptions (for example, logistic, Poisson or negative binomial for counts and rates, normal for biochemical measures, and so forth). Within each model, effect size (that is, difference between PPF-LLIN and LLIN clusters) will, as appropriate, be adjusted for clustering within the groups of villages, the shortest distance between each PPF-LLIN cluster of villages and its nearest control, LLIN, village-cluster, the time of use of current nets (to account for different times in PPF-LLIN and LLIN conditions for each group of villages), village-cluster size, sex distribution, house design, bed net use, and other relevant potential confounding factors. Effect sizes will be presented as mean differences or rate differences/ratios as appropriate, with 95% confidence intervals.

### Outcome 2 - malaria transmission

Differences in malaria transmission experienced in the 2 arms of the study will be estimated by comparing the EIR in village clusters between the 2 arms, adjusted for length of time under PPF-LLIN and distance between village clusters with the 2 different types of nets. Generalised estimating equations will be used to estimate differences in numbers of indoor mosquitoes, adjusting for repeated measures within clusters and possible covariates.

#### Secondary endpoints

For the secondary clinical endpoints we will compare: (i) mean Hb concentration in children at the end of the transmission seasons; (ii) incidence of malaria defined by history of fever or temperature ≥37.5 °C and a *P. falciparum* parasitaemia cut-off defined to give optimum sensitivity and specificity in this setting; (iii) parasite rates (sexual and asexual) in children at the end of the transmission seasons; (iv) number of children with enlarged spleens at the end of the transmission seasons; and (v) prevalence of anaemia (mild, moderate, severe). Anaemic statuses will be defined as: severe anaemia = Hb <5 g/dL, moderate anaemia = Hb range from 5 to <8 g/dL and mild anaemia = Hb range from 8 to <11 g/dL.

In general, all quantitative outcomes will be compared using appropriate summary statistics, such as mean differences or risk ratios. Hypothesis testing will be based on a 2-sample unpaired *t* test, adjusting for possible co-variates. If the data do not satisfy normality and constant variance assumptions, an appropriate non-parametric test will be applied. Both linear regression (for continuous variables such as Hb levels) and logistic regression (for dichotomous variables such as presence or absence of parasitaemia) will be used to quantify the group differences allowing for individual level covariates (such as age, house design, bed net use) using covariance structures that represent the appropriate level of clustering.

### Ethical approval

This study is conducted in accordance with the principles set forth in the International Conference on Harmonisation Harmonised Tripartite Guideline for Good Clinical Practice and the Declaration of Helsinki in its current version, whichever affords the greater protection to the participants. It was approved by the Comité d’Ethique pour la Recherche en Santé on 8 October 2013 (ref: 2013-10-101), the Institutional review board from Centre National de Recherche et de Formation sur le Paludisme and by Durham University Biological and Biomedical Sciences Devolved Ethics Sub-Committee on 17 January 2014.

## Discussion

The future of malaria vector control in sub-Saharan Africa is threatened by the rapid rise of malaria vectors resistant to pyrethroids [5], currently the only insecticide class available for treating LLINs. Finding solutions to the problem of insecticide resistance is therefore immensely important to ensure that malaria control remains effective. Here we assess whether PPF-LLIN, which contain permethrin and pyriproxyfen, provide additional protection against permethrin-resistant malaria vectors compared with current best practice of LLINs alone. The trial is designed to measure whether the double intervention will provide greater protection against clinical malaria in children by substantially (>25%) reducing transmission.

This is the first clinical trial where a combination of insecticides will be used on one bed net. Unusually the net includes pyriproxyfen which is an insect growth regulator. Unlike conventional insecticides such as permethrin, its main mode of action is not to kill mosquitoes, but to reduce their fertility and fecundity, once they have blood-fed. We hypothesise that the impact of this intervention will be greatest when a large number of people/communities use this net over a wide area. In essence we anticipate that the intervention will have a community-wide impact, reducing the number of mosquitoes within a PPF-LLIN treated area. It is largely for this reason that we have selected a novel study design where this new net is rolled out in a step-wedge fashion to the populations over a 2-year period. We expect that as coverage increases those living in clusters with PPF-LLIN surrounded by other clusters with PPF-LLIN will be better protected against malaria vectors than those on the fringes, where mosquitoes may spill-over from areas without PPF-LLIN. Thus, in the analysis we will consider both where and when people get malaria in order to estimate the efficacy of PPF-LLIN.

The main outcome measure will be clinical malaria incidence as measured by PCD which is considered to generate information of more direct relevance to public health than active case detection and is thus more suitable for evaluating population-level malaria interventions [39]. It is particularly relevant as many African countries, like Burkina Faso, have policies that malaria should be eliminated as a public health problem. The clinical outcomes will be measured in children aged 6 months to 5 years old as this age group experiences the greatest burden of malaria in this country. The lower limit of 6 months has been selected for cultural reasons and since younger children have been shown to be less likely to develop clinical malaria within their first 6 months of life [40-44].

This apparent protection has been linked to passive transfer of anti-malaria antibodies by earlier experimental studies [44-46]. In addition to quantifying malaria incidence and parasite rates, we will measure anaemia in the community surveys, since malaria is a major risk for anaemia in these populations (as other causes, such as hookworm infection, are infrequent); thus, documented changes in anaemia will complement the main outcomes.

There are no apparent risks to the safety of individuals or communities in the use of the conventional nets. Permethrin-treated long-lasting nets have been fully evaluated by the WHO Pesticide Evaluation Scheme and approved for vector control [6]. However, PPF-LLIN have not previously been evaluated in the field before, apart from small-scale experimental hut studies [11]. Whilst we do not anticipate any serious impacts of the combination of permethrin and pyriproxyfen since the safety profile of both these active compounds is deemed acceptable by the WHO Pesticide Evaluation Scheme, we cannot exclude the possibility that there might be side effects when using the combination. For this reason it is important to assess AEs and especially any SAEs in study subjects during the course of the trial to determine whether there is an excess of side effects associated with the PPF-LLIN.

Study children will benefit from a health check at the surveys and all participants will benefit from the health facility clinical services which will be supported by training and the presence of study nurses in addition to government staff. It is also important that the correct normal practice of parents/carers in the case of their child becoming sick is not altered in a detrimental fashion by their child being enrolled in the study. For example a parent/carer may delay taking a child to the nearest health facility/post if they think that a study nurse is visiting their village the next day. Both the provision of fares when a children reports sick at a health facility and emphasis of the vital role of parent/carers during enrolment and the conduct of the study will help to mitigate against this.

The rapid spread of pyrethroid resistance in African vectors is a cause of concern. Although we are currently lacking direct evidence that this is interfering with malaria control, it is likely that this will become an issue in future. There is therefore an urgent need to find alternative strategies for dealing with insecticide resistance. A net like PPF-LLIN that uses a combination of 2 different classes of active ingredients represents a possible solution to the problem since it is less likely that vectors will be resistant to both classes, especially since no cross resistance has been reported for these 2 active ingredients. The results of this trial will therefore be of interest to those involved in malaria control in Burkina Faso and other countries as an insecticide resistance management strategy.

## Trial status

Recruiting.

## Additional file

Additional file 1:
**Information sheet and consent form.**

## Abbreviations

AE: Adverse event
CDC: Centers for disease control and prevention
CRF: Case report form
CV: Coefficient of variation
EIR: Entomological inoculation rate
Hb: Haemoglobin
LLIN: Long-lasting insecticidal net
PCD: Passive case detection
PPF-LLIN: Permethrin and pyriproxyfen long-lasting insecticidal nets
RDT: Rapid diagnostic test
SAE: Serious adverse event
WHO: World health organization

## References

[1] O’Meara WP, Mangeni JN, Steketee R, Greenwood B (2010). Changes in the burden of malaria in sub-Saharan Africa. Lancet Infect Dis.

[2] WHO (2014). World Malaria Report 2014.

[3] Lengeler C (2004). Insecticide-treated bed nets and curtains for preventing malaria. Cochrane Database Syst Rev.

[4] Pluess B, Tanser FC, Lengeler C, Sharp BL (2010). Indoor residual spraying for preventing malaria. Cochrane Database Syst Rev.

[5] Ranson H, N’Guessan R, Lines J, Moiroux N, Nkuni Z, Corbel V (2011). Pyrethroid resistance in African anopheline mosquitoes: what are the implications for malaria control?. Trends Parasitol.

[6] WHO (2006). Pesticides and Their Application for the Control of Vectors and Pest of Public Health Importance.

[7] Zaim M, Aitio A, Nakashima N (2000). Safety of pyrethroid-treated mosquito nets. Med Vet Entomol.

[8] Snow RW, Rowan KM, Lindsay SW, Greenwood BM (1988). A trial of bed nets (mosquito nets) as a malaria control strategy in a rural area of The Gambia, West Africa. Trans R Soc Trop Med Hyg.

[9] Clarke SE, Bøgh C, Brown RC, Pinder M, Walraven GEL, Lindsay SW (2001). Do untreated bednets protect against malaria?. Trans R Soc Trop Med Hyg.

[10] Ohashi K, Nakada K, Ishiwatari T, Miyaguchi J, Shono Y, Lucas JR (2012). Efficacy of pyriproxyfen-treated nets in sterilizing and shortening the longevity of *Anopheles gambiae* (Diptera: Culicidae). J Med Entomol.

[11] Ngufor C, N’Guessan R, Fagbohoun J, Odjo A, Malone D, Akogbeto M (2014). Olyset Duo (R) (a pyriproxyfen and permethrin mixture net): an experimental hut trial against pyrethroid resistant *Anopheles gambiae* and *Culex quinquefasciatus* in southern Benin. PLoS One.

[12] Mbare M, Lindsay SW, Fillinger U (2014). Pyriproxifen for mosquito control: female sterilization or horizontal transfer to oviposition substrates by *Anopheles gambiae* sensu stricto and *Culex quinquefasciatus*. Parasit Vectors.

[13] Itoh T (1994). Utilization of bloodfed females of *Aedes aegypti* as a vehicle for the transfer of the insect growth regulator, pyriproxyfen, to larval habitats. Tropic Med.

[14] Hatakoshi M, Kawada H, Nishida S, Kisida H, Nakayama I (1987). Laboratory evaluation of 2 -(1-methyl-2-(4-phenoxyphenoxy) ethoxy) pyridine against larvae of mosquitoes and housefly. Jpn J Sanit Zool.

[15] Harris C, Lwetoijera DW, Dongus S, Matowo NS, Lorenz LM, Devine GJ (2013). Sterilising effects of pyriproxyfen on *Anopheles arabiensis* and its potential use in malaria control. Parasit Vectors.

[16] Sihuincha M, Zamora-Perea E, Orellana-Rios W, Stanil JD, Lopez–Sifuentes V, Vidal-Ore C (2005). Potential use of pyriproxyfen for control of *Aedes aegypti* (Diptera: Culicidae) in Iquitos. Peru. J Med Entomol.

[17] Yapabandara AM, Curtis CF (2002). Laboratory and field comparisons of pyriproxyfen, polystyrene beads and other larvicidal methods against malaria vectors in Sri Lanka. Acta Trop.

[18] Kouyate B, Sie A, Ye M, De Allegri M, Muller O (2007). The great failure of malaria control in Africa: a district perspective from Burkina Faso. PLoS Med.

[19] Pinder M, Jawara M, Jarju LBS, Kandeh B, Jeffries D, Lluberas MF (2011). To assess whether indoor residual spraying can provide additional protection against clinical malaria over current best practice of long-lasting insecticidal mosquito nets in The Gambia: study protocol for a two-armed cluster-randomised trial. Trials.

[20] Dabiré K, Diabaté A, Namountougou M, Djogbenou L, Wondji C, Chandre F, et al. Trends in insecticide resistance in natural populations of malaria vectors in Burkina Faso, West Africa: 10 years’ surveys. In: Perveen F, editor. Insecticides - Pest Engineering. 2012. On-line publication: Intech. http://www.intechopen.com/books/insecticides-pest-engineering/trends-in-insecticide-resistance-in-natural-populations-of-malaria-vectors-in-burkina-faso-west-afri.

[21] Moorthy VS, Reed Z, Smith PG (2007). Measurement of malaria vaccine efficacy in Phase III trials, report of a WHO consultation. Vaccine.

[22] Trape JF, Rogier C (1996). Combating malaria morbidity and mortality by reducing transmission. Parasitol Today.

[23] Alonso PL, Lindsay SW, Armstrong Schellenberg JRM, Gomez P, Hill AG, David PH (1993). A malaria control trial using insecticide-treated bed nets and targeted chemoprophylaxis in a rural area of The Gambia, West Africa. 2. Mortality and morbidity from malaria in the study area. Trans R Soc Trop Med Hyg.

[24] Konate AT, Yaro JB, Ouedraogo AZ, Diarra A, Gansane A, Soulama I, et al. Morbidity from malaria in children in the year after they had received intermittent preventive treatment of malaria: a randomised trial. PLoS One. 2011;6. 10.1371/journal.pone.0023391 PMC3155539 21858097

[25] Baudon DGP, Rea D, Carnevale P (1985). A study of malaria morbidity in a rural area of Burkina Faso (West Africa). Trans R Soc Trop Med Hyg.

[26] Smith T, Schellenberg JA, Hayes R (1994). Attributable fraction estimates and case definitions for malaria in endemic areas. Stat Med.

[27] Lindsay SW, Shenton FC, Snow RW, Greenwood BM (1989). Responses of *Anopheles gambiae* complex mosquitoes to the use of untreated bednets in The Gambia. Med Vet Entomol.

[28] Wirtz RA, Duncan JF, Njelesani EK, Schneider I, Brown AE, Oster CN (1989). ELISA method for detecting *Plasmodium falciparum* circumsporozoite antibody. Bull World Health Organ.

[29] Scott JA, Brogdon WG, Collins FH (1993). Identification of single specimens of the *Anopheles gambiae* complex by the polymerase chain reaction. Am J Trop Med Hyg.

[30] Fanello C, Santolamazza F, della Torre A (2002). Simultaneous identification of species and molecular forms of the Anopheles gambiae complex by PCR-RFLP. Med Vet Entomol.

[31] Bass C, Nikou D, Donnelly MJ, Williamson MS, Ranson H, Ball A (2007). Detection of knockdown resistance kdr mutations in *Anopheles gambiae*: a comparison of two new high-throughput assays with existing methods. Malar J.

[32] Jones CM, Liyanapathirana M, Agossa FR, Weetman D, Ranson H, Donnelly MJ (2012). Footprints of positive selection associated with a mutation (N1575Y) in the voltage-gated sodium channel of *Anopheles gambiae*. Proc Natl Acad Sci U S A.

[33] WHO (2013). Test Procedures for Insecticide Resistance Monitoring in Malaria Vector Mosquitoes.

[34] Smith PG, Morrow R (1996). Field Trials of Health Interventions in Developing Countries: A Toolbox.

[35] Hayes RJ, Bennett S (1999). Simple sample size calculation for cluster-randomized trials. Int J Epidemiol.

[36] Eldridge SM, Ashby D, Kerry S (2006). Sample size for cluster randomized trials: effect of coefficient of variation of cluster size and analysis method. Int J Epidemiol.

[37] Kleinschmidt I, Omumbo J, Briet O, van de Giesen N, Sogoba N, Mensah NK (2001). An empirical malaria distribution map for West Africa. Trop Med Int Health.

[38] Hawley WA, Phillips-Howard PA, ter Kuile FO, Terlouw DJ, Vulule JM, Ombok M (2003). Community-wide effects of permethrin-treated bed nets on child mortality and malaria morbidity in western Kenya. Am J Trop Med Hyg.

[39] Schellenberg D, Aponte J, Kahigwa E, Mshinda H, Tanner M, Menendez C (2003). The incidence of clinical malaria detected by active case detection in children in Ifakara, southern Tanzania. Trans R Soc Trop Med Hyg.

[40] Brabin B (1990). An analysis of malaria parasite rates in infants: 40 years after MacDonald. Trop Dis Bull.

[41] Bruce-Chwatt LJ (1952). Malaria in African infants and children in Southern Nigeria. Ann Trop Med Parasitol.

[42] Macdonald G (1950). The analysis of malaria parasite rates in infants. Trop Dis Bull.

[43] Snow RW, Nahlen B, Palmer A, Donnelly CA, Gupta S, Marsh K (1998). Risk of severe malaria among African infants: direct evidence of clinical protection during early infancy. J Infect Dis.

[44] Cohen S, Mc GI, Carrington S (1961). Gamma-globulin and acquired immunity to human malaria. Nature.

[45] Edozien JC, Gilles HM, Udeozo IOK (1962). Adult and cord-blood gamma-globulin and immunity to malaria in Nigerians. Lancet.

[46] McGregor IA, Carrington SP, Cohen S (1963). Treatment of East African *P. falciparum* malaria with West African human γ-globulin. Trans R Soc Trop Med Hyg.

