# Supplementary material for: The AvecNet Trial to assess whether addition of pyriproxyfen, an insect juvenile hormone mimic, to long-lasting insecticidal mosquito nets provides additional protection against clinical malaria over current best practice in an area with pyrethroid-resistant vectors in rural Burkina Faso: study protocol for a randomised controlled trial
Source: Trials. 2015 Mar 25;16:113. doi: 10.1186/s13063-015-0606-4 (PMC4377002; doi:10.1186/s13063-015-0606-4)
Supplement: Additional file 1: — Information sheet and consent form. [file 13063_2015_606_MOESM1_ESM.pdf]

PARTICIPANT INFORMATION AND INFORMED CONSENT AGREEMENT FORM FOR ALL PARTICIPANTS to AvecNet STUDY

Version 1.0 - 18September 2013

To assess whether addition of pyriproxyfen, an insect juvenile hormone mimic, to long-lasting insecticidal mosquito nets provides additional protection against clinical malaria over current best practice. Protocol for a two-armed cluster randomized wedge-shaped trial in Burkina Faso

Responsible scientists and medical personnel in charge of this trial	: Dr Tiono Alfred, Dr Sagnon N'Fale, Dr Margaret Pinder and Professor Steve Lindsay.
**Document type:**	**INFORMATION SHEET AND INFORMED CONSENT FORM**
**Based on:**	Protocol, v1.0, dated 18 –September- 2013
**Participant ID**	___________________________

Introduction:
You are invited to take part in a research trial. Before you decide it is important for you to understand why the research is being done. Allow me to explain what the trial is all about and what it will involve. Please take time to listen to the information carefully. Ask us if there is anything that is not clear or understood.

Purpose: To study whether a new type of long lasting insecticidal net (OLYSET DUO net) can reduce attacks of malaria in children in Burkina Faso, we invite you to participate in this research study.

What will happen: If you agree to participate, we will ask that you agree to the following activities.

1. We will provide you with a brand new insecticidal mosquito net in return for your old net. If you do not own a net we will provide you with a new insecticidal net. We ask that you use the new net for as long as possible.
2. A few weeks later we will visit your compound and ask your opinion of the net and your permission to examine the net
3. Later we will provide you with another type of net and ask you to return the net we gave you previously. Thus we will give you two nets: one with one insecticide and one with two insecticides to kill mosquitoes.
4. We think both types of nets are safe to use, but in order to check that they are we will ask that you answer a few questions about your health at the end of the first rainy season. If you are worried about the net or feel unwell we ask you to contact the Dr Sagnon N'Falé Tel: 70 23 91 09 or Dr. Alfred B. Tiono, Tel 70 28 57 26.

Duration: Your participation to this research will be 27 months
Risks: There is a lot of information that the insecticides used on the bednets are safe to people and only kill mosquitoes and insects. However, just be sure we will ask you to tell us if you have had any problem with the net.

Benefits: You will receive free bednets by participating in this study. You will be allowed to keep your net after the study has been completed. The results of the study will help us to control malaria better.

Confidentiality: Any information you provide us will be kept private. This information is usually collected on forms (paper forms or computer pages) without your name or other specific details that could be used to name you. However, sometimes your name or other specific information (i.e. date of birth) is needed to make sure that the information in the form is correct. We will keep your personal information in confidence (secret). This means we will only tell those who have a need or right to know. These people are generally sworn to secrecy and might include people who are involved in running and reviewing this study (study team and monitors, other people from the sponsor's office or governmental agencies and the Ethics Committee/Institutional Review Board). The results from this study which do not name you may be written up and given to these people. They will check that the records and results of the study are correct. This information may be stored on a computer. Signing this form means you agree to this. Wherever possible, we will only send out information that has your name and address removed so that your identity will remain confidential.

Payment: You will not receive any payment for your participation in this study.

Institutional Approval: This study has been approved by the National Ethical Committee of Burkina and the Institutional Review Board of CNRFP.

If you have questions related to your rights as a research participant, please contact The Chairman of the National Ethical committee, Tel: 50 32 41 76 and/or the Chairperson of the CNRFP IRB Tel: 70 24 06 84

INFORMED CONSENT FORM

I, ……………………………………………………………(name) do hereby consent to participating in the trial titled: AvecNet.

I have been given the opportunity to ask questions concerning this trial. Any such questions have been answered to my full satisfaction. Should any further questions arise concerning this trial I may contact Dr Sagnon N'Falé Tel: 70 23 91 09 or Dr. Alfred B. Tiono, Tel 70 28 57 26

I also understand that I may revoke this consent at any time without penalty or loss of benefits, if any.

Thumbprint if unable to sign

Signature/Thumbprint of participant

....Date………………………

Address ………………………………….

………………………………….

………………………………….

Witness name…………………………………………………………………………………….

Witness signature…………………………………………………… Date………………………………….

Investigator's name:………………………………………………………………………….

Signature:…………………………………………………… Date……………………………………..
